# GEA 3162, a peroxynitrite donor, induces Bcl-2-sensitive, p53-independent apoptosis in murine bone marrow cells

**DOI:** 10.1016/j.bcp.2007.06.028

**Published:** 2007-06-23

**Authors:** Emma L. Taylor, John T. Li, Joan C. Tupper, Adriano G. Rossi, Robert K. Winn, John M. Harlan

**Affiliations:** a Institute of Biomedical and Clinical Science, Peninsula Medical School, Universities of Exeter and Plymouth, St Luke's Campus, Heavitree Rd, Exeter, Devon EX1 2LU, UK; b Department of Anesthesia/Critical Care Medicine, Children's Hospital Los Angeles, University of Southern California Keck School of Medicine, Los Angeles, CA 90027, USA; c Department of Medicine, Research and Training Building, University of Washington, Harborview Medical Center, 300 Ninth Ave, Seattle, WA 98104, USA; d Centre for Inflammation Research, Queens Medical Research Institute, University of Edinburgh, 47 Little France Crescent, Edinburgh, EH16 4TJ, UK; e Department of Surgery, Research and Training Building, University of Washington, Harborview Medical Center, 300 Ninth Ave, Seattle, WA 98104, USA

**Keywords:** Peroxynitrite, Apoptosis, Caspase, p53, Bcl-2, Myeloid

## Abstract

Apoptosis may be regulated by oxidants such as peroxynitrite (ONOO^−^). The tumour suppressor, p53, has been reported to play a crucial role in apoptosis induced by oxidants, therefore we assessed the ability of a ONOO^−^ donor, GEA 3162, to activate caspases and induce mitochondrial permeability in a p53-deficient murine bone marrow cell line, Jaws II. Furthermore, these cells were stably transfected with Bcl-2, in order to investigate the impact of this survival protein on ONOO^−^-induced apoptosis. GEA 3162 activated caspases and induced loss of mitochondrial membrane potential in Jaws II cells. In particular, caspases 3 and 2 were activated, alongside minor activation of caspases 8 and 9, and apoptosis was partially dependent upon p38 MAP kinase activation, with little or no role for JNK. Overexpression of Bcl-2 abolished activation of all caspases and reduced the change in mitochondrial membrane potential. Thus, we have demonstrated that the ONOO^−^ donor, GEA 3162, induces apoptosis in Jaws II murine myeloid cells despite lacking functional p53, via a pathway that principally involves caspases 2 and 3 and mitochondrial changes. This is blocked by overexpression of Bcl-2 via a mechanism that does not appear to merely reflect stabilisation of the mitochondrial membrane.

## 1. Introduction

Apoptosis is a physiological form of programmed cell death that plays important roles in embryogenesis, inflammation and the safe and efficient clearance of damaged or unwanted cells. This process is characterised by cellular and nuclear condensation, maintenance of cell membrane integrity, the exposure of recognition molecules for phagocytic removal and internucleosomal DNA fragmentation [1]. During apoptotic cell death, a range of cysteine proteases called caspases are necessarily activated, which cleave proteins after aspartic acid residues following activation from their zymogen forms [2].

There are two principal pathways for the execution of apoptosis; a death receptor pathway and a stress pathway, as reviewed by Wyllie [1], and Zimmermann et al. [3]. The extrinsic death receptor-mediated pathway involves engagement of ligands such as tumour necrosis factor-α (TNF-α) or Fas ligand (FasL) with the appropriate receptor and proceeds through activation of caspase 8 (initiator caspase) and caspase 3, which is critical in cleaving cellular components to drive the irreversible dismantling of the cell. Additionally, the mitochondrial pathway described below can be activated through death receptor ligation via translocation of the truncated (active) form of Bid (tBid) to the mitochondria following cleavage by caspase 8, as an amplification mechanism.

The intrinsic pathway occurs in response to cellular damage or external stresses, and mitochondria play a central role in this pathway. Activation of the stress pathway leads to mitochondrial changes, in which the membrane potential is lost, causing mitochondrial permeability. This allows the loss of pro-apoptotic factors from the mitochondria, formation of an ‘apoptosome’, and activation of caspase 9 (initiator caspase) and ultimately caspase 3. These mitochondrial changes are tightly regulated by the balance of pro- and anti-apoptotic members of the Bcl-2 proto-oncogene family [4], which interact with each other and the mitochondrial membrane to either promote or prevent mitochondrial permeabilisation. Pro-apoptotic members of the Bcl-2 family include Bax, Bad, Bid and Bak and tend to be located within the cytosol, whereas anti-apoptotic members such as Bcl-2, Bcl-_XL_ and A1 are predominantly found on the mitochondrial membrane itself.

Mitochondrial changes in response to cellular damage or stress are often preceded by activation of the transcription factor, p53. This tumour suppressor induces apoptotic cell death if DNA damage is severe or irreparable. It controls transcription levels of several proteins involved in apoptosis in order to push the balance towards cell death, notably by increasing the pro-apoptotic proteins Fas (CD95), Bax, Bid, Noxa and Puma [4,5] and down-regulating expression of the anti-apoptotic protein, Bcl-2 [6].

Further molecules with a role in the regulation of apoptosis are the mitogen activated protein (MAP) kinases. The JNK and p38 MAP kinase families are involved in the cellular response to stress and are important in apoptosis [7]. However, the relationship between pro- and anti-apoptotic effects of these kinases is complex, and may be cell type or stimulus-specific [8].

Peroxynitrite (ONOO^−^) is a powerful oxidant species formed through the combination of NO and superoxide anion (O_2_^−^). Inflammatory cells are able to produce both species when activated [9], and ONOO^−^ is thought to have bactericidal effects in acute inflammation, and has additionally been shown to promote apoptosis in several cell types [10,11].

Previous studies have demonstrated that the ONOO^−^ donor, GEA 3162, is able to promote apoptotic cell death in neutrophils [12-14] in a caspase-dependent manner. However, the effects of this compound on apoptosis have not been investigated in other cells of the myeloid lineage. We set out to investigate the effects of the ONOO^−^ donor, GEA 3162, on apoptosis in the Jaws II murine immature bone marrow cell line, which is derived from the p53−/− mouse. We hypothesised that GEA 3162 would initiate apoptosis in these cells despite their lack of p53, and that overexpression of the survival protein, Bcl-2, would abolish this response through stabilisation of the mitochondrial membrane.

## 2. Materials and methods

### 2.1. Cell culture

Immature mouse bone marrow cells (Jaws II; ATCC, Manassas, VA) [15] were cultured in alpha minimum essential medium with ribonucleosides, deoxyribonucleosides enriched with 20% heat-inactivated fetal bovine serum (Hyclone Laboratories, Logan, UT), l-glutamine 4 mM, and sodium pyruvate 1 mM (all purchased from BioWhittaker, Inc., Walkersville, MD) in the presence of 5 ng/ml GM-CSF (R&D Systems, Inc., Minneapolis, MN). Cells were seeded into T75 culture flasks. Flasks were incubated at 37 °C in a humidified atmosphere containing 5% CO_2_ until approximately 70–80% confluence was achieved. Cells were passaged 1 in 4 every 3–4 days.

In order to harvest the cells, suspension cells were removed into 50 ml centrifuge tubes before addition of trypsin-EDTA to the flasks. Cells were incubated until the adherent cells detached and were removed into the appropriate centrifuge tube, and cell numbers counted using a haemacytometer. The tubes were centrifuged (1000 rpm, 10 min) to form a cell pellet, then supernatant was aspirated and the cells resuspended in Jaws II medium at a density of 5 × 10^5^ cells/ml before being plated out as required.

### 2.2. Construction and stable expression of Bcl-2 cDNA

cDNA encoding the intronless open reading frame of the 717 base pair human Bcl-2α (pORF-hBcl-2) (InvivoGen, San Diego, CA) was cloned into shuttle plasmid SL1180 (Amersham Pharmacia Biotech, Piscataway, NJ) utilizing the Nco I and Nhe I (New England BioLabs, Inc., Beverly, MA) restriction enzyme sites. The pORF-hBcl-2 was subsequently cloned into the EcoR I and Xho I (New England BioLabs, Inc., Beverly, MA) sites on the multiple cloning region of the bicistronic retroviral expression plasmid, pBMN-IRES-enhanced green fluorescent protein (EGFP) (kindly provided by Dr. Gary Nolan, Stanford University, Palo Alto, CA) [16]. High titer second-generation helper free retrovirus was produced by calcium phosphate mediated transfection of the Phoenix ecotropic packaging cell line (ATCC, Manassas, VA) with either 24 μg of the hBcl-2 expression plasmid or pBMN-IRES-EGFP control plasmid. Recombinant retroviral supernatant was collected 48 h after transfection and filtered through a Millex-HV 0.45 μm filter (Millipore Corp., Bedford, MA). For transduction, cell culture media from ∼70% confluent JAWS II cells in 6-well plates (Corning Inc., Corning, NY) were replaced with 2.5 ml of retrovirus supernatant and centrifuged for 2 h (1430 × g at 32 °C) and then incubated for 10 h (5% CO_2_, 37 °C). Upon completion of the incubation period, retroviral supernatant was replaced by appropriate normal growth medium for each cell type. Cells were sorted for stable retrovirus transfection based on EGFP expression using a FACVantage SE cell sorter (Beckton Dickinson Corp., Franklin Lakes, NJ). High-expressing cells were collected and used in subsequent experiments, as previously published [17].

### 2.3. Bcl-2 western blot

JAWS II-Bcl-2 or JAWS II-GFP cells were lysed in 60 mm tissue culture dishes (Becton Dickinson, Franklin Lakes, NJ) using ice-cold modified radioimmunoprecipitation (RIPA) buffer (Tris–HCl 50 mM, NP-40 1%, Na-deoxycholate 0.25%, NaCl 150 nM, Na_3_VO_4_ 1 mM, NaF 50 mM) (all purchased from Sigma, St. Louis, MO) with one Complete Mini™ tablet (Roche, Indianapolis, IN) per 10 ml of modified RIPA buffer. Cell debris was removed by centrifugation at 14,000 × g for 15 min at 4 °C. Supernatant was collected and stored at −20 °C. BCA Protein Assay kit (Pierce, Rockford, IL) was used to measure protein concentrations of cell lysates. Cell lysates, 20 μg total protein per lane, were resolved on Novex® 4–20% Tris–glycine gels (Invitrogen, Carlsbad, CA) and transferred onto methanol activated Immobilon™ PVDF membrane (Millipore Co., Bedford, MA). Membrane was blocked with 5% milk (Western Family Foods, Portland, OR) in TBS (Sigma, St. Louis, MO) at 4 °C overnight. After blocking, membrane was probed with mouse anti-human Bcl-2 monoclonal antibody (#610539 BD Transduction Labs, San Diego, CA) at a 1:1000 dilution in TBS with 1% tween-20 (TBST) (Sigma, St. Louis, MO) for 1 h at room temperature. Membrane was washed three times in TBST. Goat anti-mouse IgG HRP conjugated secondary antibody (#610094 BD Transduction Labs, San Diego, CA) was applied at 1:10,000 dilution in TBST and incubated for 1 h at room temperature. Membrane was washed four times in TBST followed by two washes in TBS. Enhanced chemiluminescence (ECL) plus (Amersham, Buckinhamshire, England) was applied for 5 min and chemiluminescence was detected on radiographic film (Eastman Kodak Co., Rochester, NY).

### 2.4. MTT assay

A colorimetric assay using (3-(4,5-dimethylthiazol-2-yl)-2,5-diphenyltetrazolium bromide (MTT) provides a measure of the cytotoxicity of agents. The mitochondria of viable cells metabolise MTT to turn the solution from yellow to purple, which can be measured using a spectrophotometer at a wavelength of 500–600 nm.

In order to measure cell viability following exposure to GEA 3162 (30–100 μM), 20,000 cells per well (40 μl) were plated into a 96-well plate, which was then incubated at 37 °C overnight, before addition of 50 μl medium. GEA 3162 (10 μl of 10× stock) was added to final concentrations as shown in the figure legends (concentrations that have previously been shown to promote apoptosis in human neutrophils [18]) and the plate incubated for 4 h. The MTT assay was then carried out according to the manufacturers' instructions. Briefly, 10 μl of MTT solution was added to each well and the plate incubated at 37 °C for 4 h before addition of 100 μl of solubilisation solution. The plate was then read with a test wavelength of 570 nm and a reference wavelength of 630 nm.

### 2.5. Homogeneous caspase activation

Caspase proteases are activated during apoptotic cell death. Fluorogenic substrates can be cleaved by caspases, giving a quantitative indication of caspase enzyme activity which can be measured by fluorescent plate reader.

In order to measure levels of caspase activation in Jaws II cells treated with GEA 3162 (Alexis Biochemicals, San Diego, CA) or the pan-kinase inhibitor, staurosporine (STSP; Sigma, St. Louis, MO), 20,000 cells per well (40 μl) were plated into an opaque 96-well plate, which was then incubated at 37 °C overnight. GEA 3162 or STSP (10 μl of 10× stock) were added to final concentrations as shown in the figure legends (concentrations that have previously been shown to promote apoptosis in human neutrophils [18]) in the absence or presence of the pan-caspase inhibitor, zVAD (100 μM). The volume in each well was made up to 100 μl with 50 μl culture medium. Following 4 h incubation at 37 °C (time point determined by preliminary time course studies; data not shown), caspase activation was measured using a homogeneous caspase detection kit (Roche, Indianapolis, IN) according to the manufacturer's instructions. Briefly, 100 μl of caspase substrate was added to each well, plus two wells containing positive control lysates provided in the kit, and two wells containing medium alone as ‘blank’ wells. The plate was then incubated at 37 °C for at least 1 h before fluorescence was read using a fluorescent plate reader with excitation at 485 nm and emission measured at 530 nm. Background fluorescence from ‘blank’ wells was then subtracted from all values obtained.

The same protocol was followed for other experiments using the pharmacological inhibitors, SP600125 (JNK inhibitor) and SB203580 (p38 inhibitor). For these experiments, the inhibitor was diluted in Jaws II culture medium at 2× desired concentration before addition of 50 μl to the wells at a final concentration of 10–50 μM.

### 2.6. Specific caspase activation

Individual caspases 2, 3, 8 and 9 were also assessed for activity using a fluorescent plate assay, in order to investigate possible pathways through which GEA 3162-induced apoptosis may proceed.

Jaws II-GFP and Jaws II–Bcl-2 cells were harvested as described above. 2 million cells were seeded into T25 flasks in 4.5 ml culture medium and incubated overnight at 37 °C. The following day, 500 μl of medium (controls) or GEA 3162 (final concentration 30–100 μM) were added, and flasks were incubated for 4 h at 37 °C. Cells were then harvested and centrifuged (1000 rpm, 5 min). Activity of caspases 2, 3, 8 and 9 was then assessed using the ApoAlert Caspase Profiling kit (Clontech, Mountain View, CA) according to the manufacturers' instructions. Briefly, cell pellets were resuspended in 400 μl of ice-cold cell lysis buffer and incubated on ice for 10 min. Following lysis, tubes were vortexed and 50 μl of cell lysate added in duplicate to wells containing immobilised substrate for individual caspases plus 50 μl of 2× reaction buffer. The plate was then covered and incubated at 37 °C for 2 h. Cleavage of caspase substrates was then measured by fluorescent plate reader, with excitation at 360 nm and emission measured at 460 nm.

### 2.7. Mitochondrial membrane permeability assay

A potential difference exists across the mitochondrial membrane in viable cells. However, during apoptosis that proceeds through the stress or mitochondrial pathway, the potential difference is lost and the mitochondria become permeable. This loss of potential difference can be measured by the use of a fluorescent dye that accumulates in the mitochondria of viable cells and fluoresces red, but is unable to enter the mitochondria of apoptotic cells and fluoresces green in the cytosol. The ratio of red:green fluorescence gives an indication of the proportion of viable:apoptotic cells, and can be measured using a fluorescent plate reader.

The change in mitochondrial membrane potential during apoptosis in Jaws II cells treated with GEA 3162 or STSP was measured using the Mit-E-ψ apoptosis detection kit (Biomol International, Plymouth Meeting, PA) according to the manufacturer's instructions. Cells were harvested as described above and 10,00,000 cells added per well of a 6-well plate in a total volume of 2 ml. Following overnight incubation at 37 °C to allow cells to adhere, culture medium (control), STSP or GEA 3162 were added to the wells to final concentrations shown in the figure legends, then cells were incubated for a further 4 h. The cells were then harvested and tubes were centrifuged (1000 rpm, 5 min) to pellet the cells. Supernatant was then aspirated and cells were resuspended in 1 ml Mit-E-ψ solution and incubated at 37 °C for 15 min. Assay buffer (2 ml) was then added and the tubes centrifuged (1000 rpm, 5 min) before resuspension of the cell pellet in 1 ml assay buffer and further centrifugation (1000 rpm, 5 min) to wash the cells. Cells were resuspended once more in 1 ml assay buffer before duplicate aliquots of 100 μl were transferred from each tube to an opaque 96-well plate, which was then analysed by fluorescent plate reader with excitation at 485 nm and emission measured at 590 nm (red fluorescence) and 530 nm (green fluorescence). The ratio of red to green fluorescence was then calculated.

### 2.8. Statistical analysis

Each experiment was carried out using duplicate samples for a minimum of three replicates, and the mean and standard error of the combined data was calculated. Significance between results was analysed using repeated measures ANOVA. Student–Newman–Keuls post-test was used to determine significance differences between treatments. A *p*-value < 0.05 was considered statistically significant.

## 3. Results

### 3.1. Characterisation of the Bcl-2 overexpressing Jaws II cell line

**Fig. 1:**
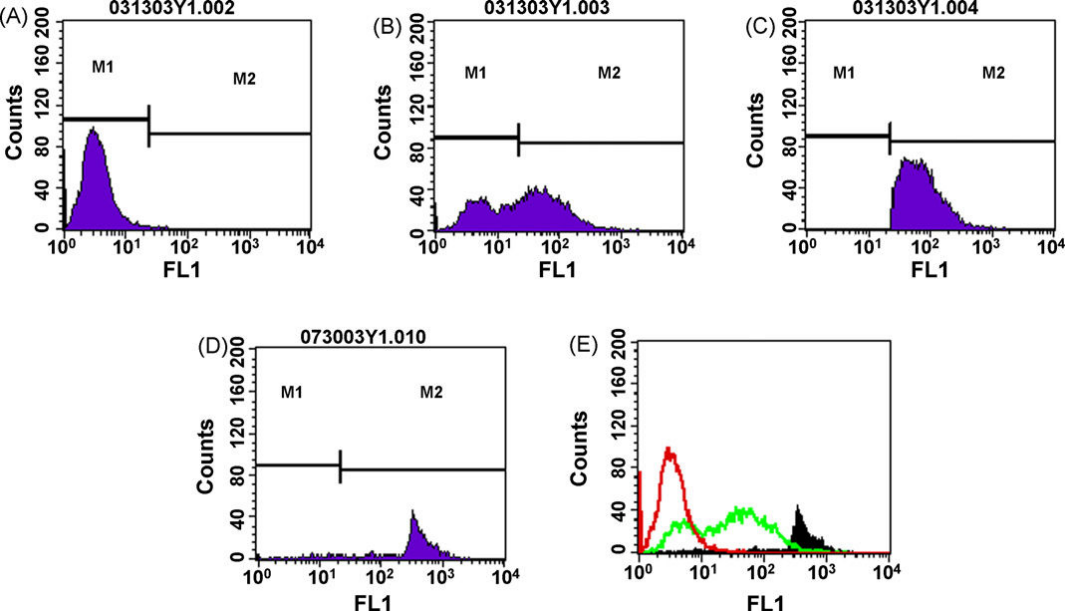
FACS analysis for transfection of JAWSII cells with retrovirus containing bcl-2-IRES-GFP. Cells successfully transfected to overexpress Bcl-2 were selected based on linked GFP expression in FL1. In panel (A) wild type cells demonstrated minimal fluorescence. (B) Cells transfected with Bcl-2-IRES-GFP retrovirus showed bimodal fluorescence and GFP positive cells were sorted based on gate M2. Post-sort analysis (C) showed a wide range in fluorescent intensity and these cells were sorted again for the highest GFP expressors (D). Panel (E) shows the population of cells after several passages: wild type (black line), transfected cells (grey line), high-expressing cells (black area).

Jaws II cells transfected with a vector encoding GFP/Bcl-2 were sorted for high-expressing cells on the basis of their green fluorescence (FL1). Non-transfected cells exhibited low fluorescence (Fig. 1A), which was shifted to a bimodal fluorescence profile following retroviral transfection (Fig. 1B). The higher expressing cells, corresponding to gate M2, were collected. Analysis of these cells showed a wide spread of fluorescence (Fig. 1C), so they were once again sorted for the highest expressing cells (Fig. 1D) which were collected and cultured for use in these studies.

**Fig. 2:**
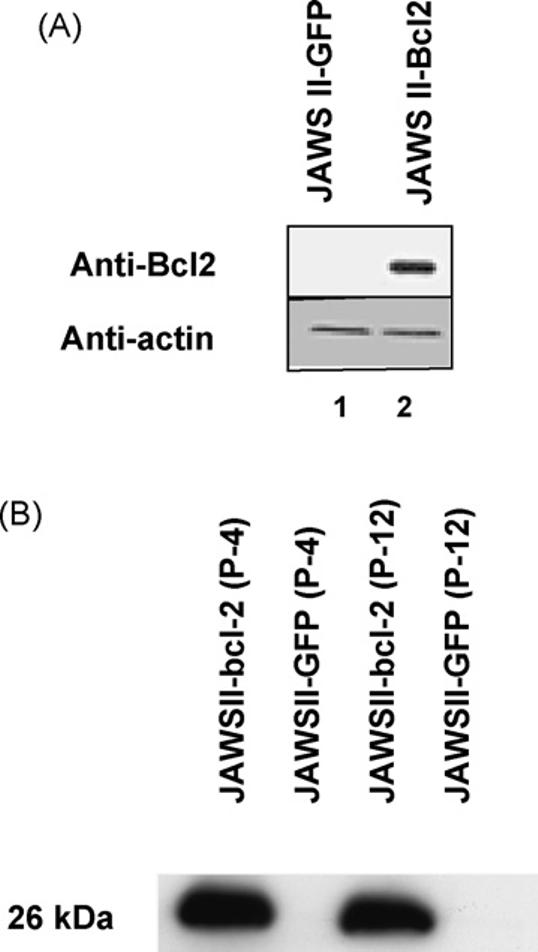
Transfection of Jaws II cells with human Bcl-2. (A) Western blot of cell lysates prepared from Jaws II cells transfected to overexpress Bcl-2 (Jaws II-Bcl-2) or vector alone (Jaws II-GFP), showing expression of Bcl-2 in Jaws II-Bcl-2 but not Jaws II-GFP cells, and equal lane loading using actin. (B) Bcl-2 overexpression was stable over multiple passages. Western blot of different passages of JAWS II cells stably transfected to overexpress human Bcl-2 or GFP. Human Bcl-2 protein levels were unchanged from passage 4 to passage 12.

Western blot analysis was carried out to demonstrate the effectiveness of the transfection of Bcl-2 into the Jaws II cell line. These blots clearly show that Jaws II-Bcl-2 cells express large quantities of Bcl-2 protein, that are not detectable in cells containing GFP vector alone (Fig. 2A, lanes 1 and 2). The blot was stripped and reprobed for actin to demonstrate that lane loading was equal for both Jaws II-GFP and Jaws II-Bcl-2 cells. Expression was stable and did not diminish over time (Fig. 2B), as cell lysates generated at both passage 4 (lanes 1 and 2) and at passage 12 show marked Bcl-2 expression in Jaws II-Bcl-2 cells but not in Jaws II-GFP cells (lanes 3 and 4).

### 3.2. Effects of GEA 3162 on cell viability in Jaws II-GFP and Jaws II-Bcl-2 cells

**Fig. 3:**
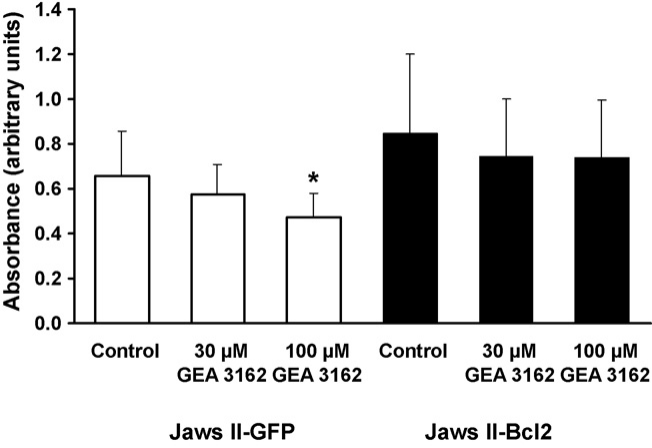
Effect of GEA 3162 on Jaws II cell viability. GFP or Bcl-2-overexpressing Jaws II cells were exposed to GEA 3162 (30–100 μM) for 4 h before assessment of cell viability by MTT assay. Data represents mean ± S.D. following *n* = 2 experiments. Asterisks represent significant (*p* < 0.05) difference from control (untreated) cells for each cell type (repeated measures ANOVA with Student–Newman–Keuls post-test).

Assessment of Jaws II cell viability by MTT assay following exposure to GEA 3162 (30 or 100 μM) for 4 h showed a decrease in viability in Jaws II-GFP cells (Fig. 3), with a significant (*p* < 0.05) effect seen with the higher concentration. Jaws II-Bcl-2 cells also showed a slight decrease in viability, although this effect was reduced compared to the control cells and was associated with more variability in absorbance measured. Both sets of cells continued to exclude Trypan Blue following treatment with GEA 3162 (data not shown).

### 3.3. Total caspase activation in response to GEA 3162 in Jaws II-GFP and Jaws II-Bcl-2 cells

**Fig. 4:**
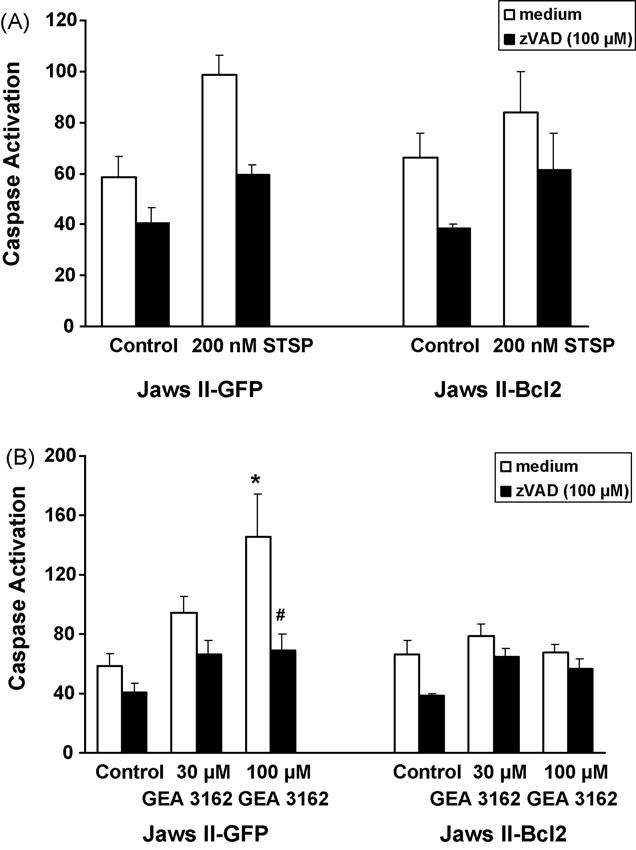
GEA 3162 induces caspase activation in Jaws II-GFP but not Jaws II-Bcl-2 cells. GFP or Bcl-2-overexpressing Jaws II cells were exposed to (A) STSP (200 nM) or (B) GEA 3162 (30–100 μM) for 4 h before assessment of homogeneous caspase substrate cleavage (white bars). As an internal control for the assay, cells were also incubated with the pan-caspase inhibitor, zVAD (100 μM; black bars). Data represents mean ± S.E.M. following *n* = 4 experiments performed in duplicate. Asterisks represent significant (*p* < 0.05) difference from control (untreated) cells for each cell type, and hashes represent significant differences in fluorescence in the absence and presence of zVAD for a given treatment (repeated measures ANOVA with Student–Newman–Keuls post-test).

Measurement of caspase activation in Jaws II cells showed enhanced activation in cells containing the GFP vector in response to GEA 3162. Following 4 h incubation in medium alone, Jaws II-GFP cells and the Bcl-2-overexpressing cells showed no significant difference in the basal level of fluorescence. In the presence of STSP (200 nM), however, fluorescence was increased (*p* > 0.05 compared with GFP control) in Jaws II-GFP cells (Fig. 4A). Overexpression of Bcl-2 reduced the increase in fluorescence in response to STSP (*p* > 0.05), although fluorescence did not return to control levels. In the presence of the pan-caspase inhibitor, zVAD (100 μM), fluorescence levels were similar in GFP cells, GFP cells plus STSP (*p* > 0.05), Bcl-2 overexpressing cells (*p* > 0.05) and Bcl-2 cells plus STSP (*p* > 0.05). Although the presence of zVAD was insufficient to return fluorescence back to control levels, it did markedly reduce the level of caspase activation in response to STSP in both Jaws II-GFP and Jaws II-Bcl-2 cells, although this was not significantly different from STSP-treated cells in the absence of zVAD (*p* > 0.05).

When Jaws II cells were exposed to the ONOO^−^ donor, GEA 3162 (30–100 μM), for 4 h, caspase activation increased in a concentration-dependent manner in Jaws II-GFP cells (Fig. 4B), with those treated with 100 μM GEA 3162 producing a statistically significant (*p* < 0.05) degree of activation. In contrast, in the presence of 100 μM zVAD, the increase in fluorescence was abrogated, and no significant difference was seen between treated and untreated cells (*p* > 0.05).

Overexpression of Bcl-2 reduced the caspase activation seen in Jaws II cells on exposure to GEA 3162. Treatment of these cells with GEA 3162 produced no significant difference in fluorescence from control (untreated) cells (*p* > 0.05). There was, however, a significant decrease in caspase activation levels in Bcl-2-overexpressing cells exposed to 100 μM GEA 3162, compared to cells containing the GFP vector alone under the same conditions (*p* < 0.05). Addition of zVAD (100 μM) had little effect in Jaws II-Bcl-2 cells, but reduced fluorescence levels very slightly (*p* > 0.05), demonstrating that the vast majority of caspase activity was already inhibited by the overexpression of Bcl-2.

### 3.4. Involvement of mitochondrial permeabilisation in apoptosis

**Fig. 5:**
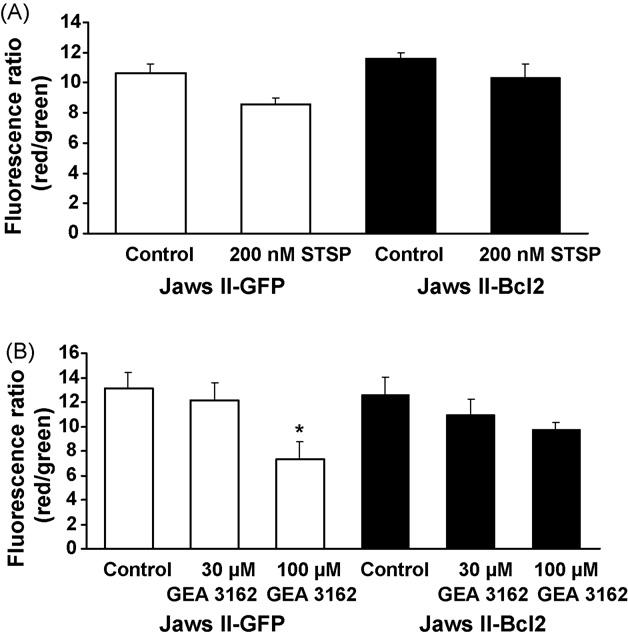
GEA 3162 induces loss of mitochondrial membrane potential that is reduced by Bcl-2 overexpression. GFP or Bcl-2-overexpressing Jaws II cells were exposed to (A) STSP (200 nM) or (B) GEA 3162 (30–100 μM) for 4 h before assessment of mitochondrial permeability. Cells were harvested and centrifuged and cell pellets resuspended and incubated in a solution of fluorescent dye before washing, then red and green fluorescence was measured. The ratio of red (intact mitochondrial membrane potential) to green (mitochondrial membrane potential lost) cells is indicative of the extent of mitochondrial-dependent apoptosis. Data represents mean ± S.E.M. following *n* = 4 experiments performed in duplicate. Asterisks represent significant (*p* < 0.05) difference from control (untreated) cells for each cell type (repeated measures ANOVA with Student–Newman–Keuls post-test).

The loss of mitochondrial membrane potential (*ψ*_M_) that is characteristic of the stress pathway of apoptosis was measured through the use of a fluorescent dye. Viable cells fluoresce red and apoptotic cells fluoresce green, therefore the red:green fluorescence ratio decreases with increasing levels of apoptotic cell death. STSP (200 nM, 4 h) promoted the loss of *ψ*_M_ in Jaws II cells containing the GFP vector alone, while overexpression of Bcl-2 caused a reduction in the change of mitochondrial potential (Fig. 5A), thereby providing a protective effect against mitochondrial permeabilisation.

In Jaws II-GFP cells exposed to GEA 3162 (30–100 μM) for 4 h, there was a concentration-dependent reduction in the fluorescence ratio compared to control cells (Fig. 5B), with exposure to 100 μM GEA 3162 producing a significant level of mitochondrial permeability (*p* < 0.05). Again, the overexpression of Bcl-2 in Jaws II cells conferred some protection against the loss of *ψ*_M_, although this was not complete. Cells exposed to 30 or 100 μM GEA 3162 were not significantly different from control (*p* > 0.05).

### 3.5. Activation of specific caspases

**Fig. 6:**
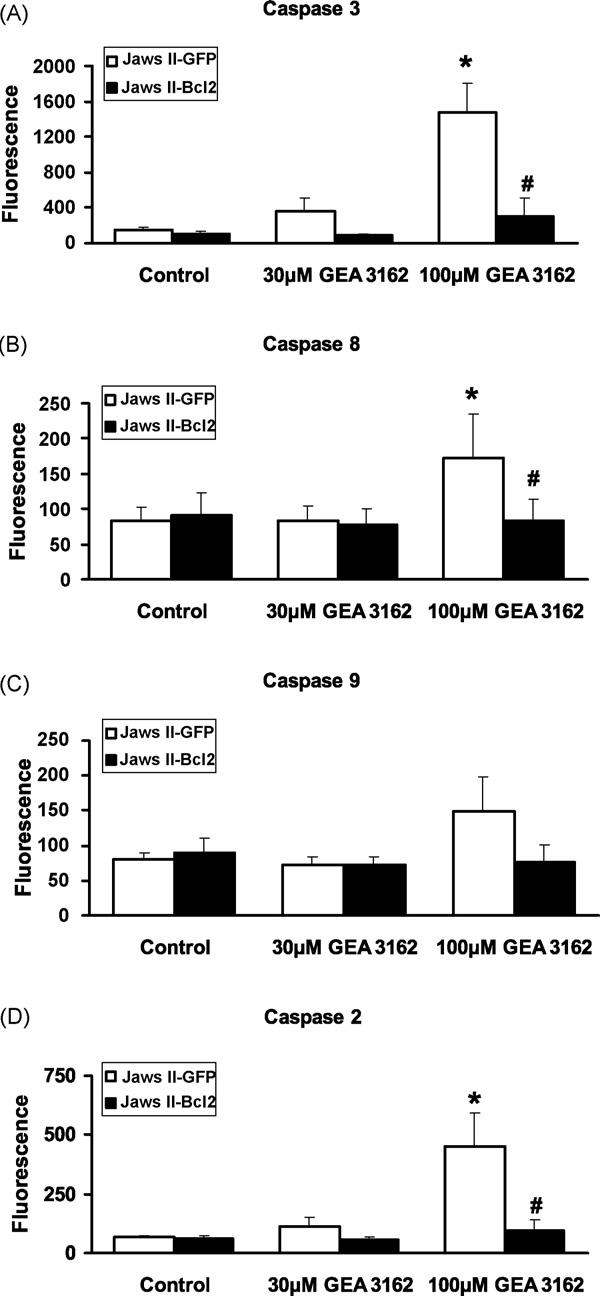
Specific caspases activated by GEA 3162. GFP or Bcl-2-overexpressing Jaws II cells were exposed to GEA 3162 (30–100 μM) for 4 h before assessment of the cleavage of specific fluorogenic substrates for (A) caspase 3, (B) caspase 8, (C) caspase 9 and (D) caspase 2. Cells were harvested, centrifuged and lysed, then lysates incubated with specific caspase substrates immobilised on a plate, with fluorescence indicating the extent of caspase activation. Data represents mean ± S.E.M. following *n* = 4 experiments performed in duplicate. Asterisks represent significant (*p* < 0.05) difference from control (untreated) cells for each cell type, and hashes represent significant differences between fluorescence in GFP and Bcl-2 cells for a given treatment (repeated measures ANOVA with Student–Newman–Keuls post-test).

Analysis of specific caspase activation showed that exposure of Jaws II-GFP cells to GEA 3162 leads to a large increase in the activity of caspases 2 and 3, and a smaller increase in caspases 8 and 9 (Fig. 6). Treatment with 30 or 100 μM GEA 3162 produced a concentration-dependent increase in caspase 3 substrate cleavage in cells transfected with the GFP vector alone, with fluorescence levels reaching approximately 230% (*p* > 0.05) and 950% (*p* < 0.05) of control levels respectively. Similarly, relatively high levels of activation were seen with caspase 2, with 30 μM GEA 3162 producing around 160% (*p* > 0.05) and 100 μM producing 650 % (*p* < 0.05) of control fluorescence. However, caspase 8 activation was less pronounced, with fluorescence in response to 30 μM GEA 3162 being virtually identical to control (*p* > 0.05), and 100 μM measured at 200% (*p* < 0.05) of control. Caspase 9 levels were virtually identical in control and 30 μM GEA 3162-treated cells (*p* > 0.05) while 100 μM-treated cells showed 185% (*p* > 0.05) of control fluorescence. In contrast, overexpression of Bcl-2 greatly reduced or abolished all GEA 3162-induced increases in fluorescence, and there was no significant difference between any treatments in these cells for any of the caspases measured, indicating that Bcl-2 overexpression prevents up-regulation of the activity of multiple caspases in response to ONOO^−^.

### 3.6. Effects of MAP kinase inhibitors on total caspase activity in Jaws II-GFP cells

**Fig. 7:**
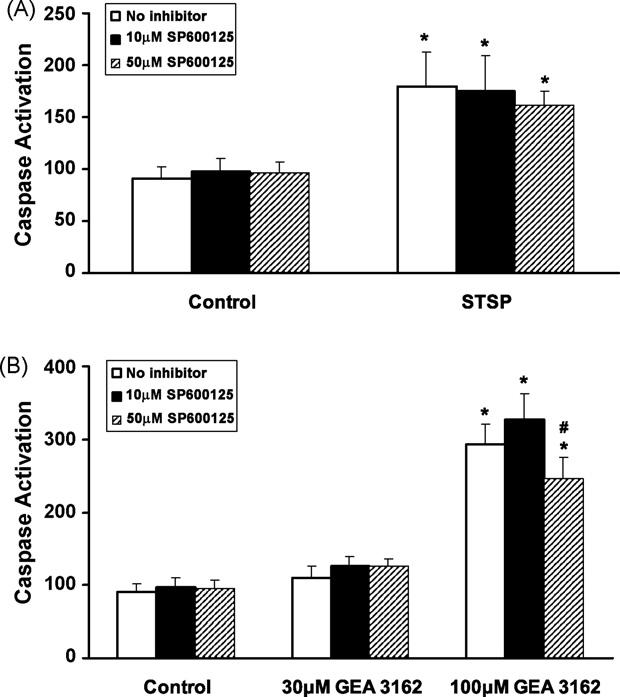
Effect of JNK inhibition on GEA 3162-induced apoptosis in Jaws II-GFP cells. Cells were exposed to (A) STSP (200 nM) or (B) GEA 3162 (30–100 μM) in the absence or presence of the JNK inhibitor, SP600125 (10–50 μM). Total caspase activity was then measured by incubation with fluorogenic caspase substrate for 1 h. Data represents mean ± S.E.M. following *n* = 5 experiments performed in duplicate. Asterisks represent significant (*p* < 0.05) difference from control (untreated) cells, and hashes represent significant differences in fluorescence in the absence and presence of SP600125 (repeated measures ANOVA with Student–Newman–Keuls post-test).

Homogeneous caspase activity in the presence of JNK (SP600125; 10–50 μM) and p38 (SB203580; 10–50 μM) MAP kinase inhibitors was also measured in Jaws II-GFP cells exposed to GEA 3162 (30–100 μM) for 4 h. SP600125 afforded slight protection against GEA 3162-induced apoptosis at the highest concentration (Fig. 7B). The level of fluorescence in untreated (control) cells was unaffected by the presence of 10 μM (*p* > 0.05) or 50 μM (*p* > 0.05) of the JNK inhibitor. The lower (30 μM) concentration of GEA 3162 caused a small increase in caspase activity although this was not statistically significant by repeated measures ANOVA (*p* > 0.05). In the presence of SP600125, fluorescence was not significantly altered. Exposure to 100 μM GEA 3162 for 4 h caused a significant increase in caspase activity (*p* < 0.05). Addition of 10 μM SP600125 caused a small increase in caspase activity (*p* < 0.05 compared to untreated cells), but addition of 50 μM SP600125 caused a small but statistically significant decrease. While this was still significantly different from untreated control cells, it was also a significant decrease compared to cells exposed to GEA 3162 in the absence of the JNK inhibitor (*p* < 0.05).

In contrast, SP600125 failed to affect caspase activation in response to 200 nM STSP (Fig. 7A). STSP-treated cells exhibited a significant increase in fluorescence compared to control (*p* < 0.05) which was not abrogated by the presence of SP600125, as fluorescence remained significantly different from control.

**Fig. 8:**
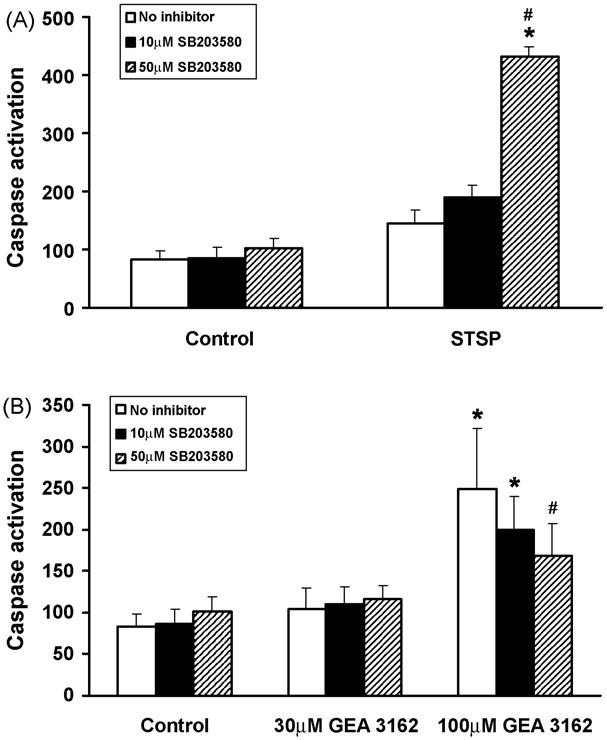
Effect of p38 inhibition on GEA 3162-induced apoptosis in Jaws II-GFP cells. Cells were exposed to (A) STSP (200 nM) or (B) GEA 3162 (30–100 μM) in the absence or presence of the p38 inhibitor, SB203580 (10–50 μM). Total caspase activity was then measured by incubation with fluorogenic caspase substrate for 1 h. Data represents mean ± S.E.M. following *n* = 3 experiments performed in duplicate. Asterisks represent significant (*p* < 0.05) difference from control (untreated) cells, and hashes represent significant differences in fluorescence in the absence and presence of SB203580 (repeated measures ANOVA with Student–Newman–Keuls post-test).

Analysis of total caspase activation in the presence of the p38 inhibitor, SB203580 (10–50 μM) showed protection of Jaws II-GFP cells from GEA 3162-induced apoptosis (Fig. 8B). The inhibitor had no effect on apoptosis in untreated (control) cells, with fluorescence at similar levels in the presence of 0, 10 and 50 μM SB203580 (*p* > 0.05 between conditions). Caspases were activated in response to 30 μM GEA 3162 (*p* > 0.05 compared to untreated cells), and addition of 10 or 50 μM of the p38 inhibitor had no effect on caspase activity (*p* > 0.05 versus no inhibitor). Increased caspase activity was also detected on exposure to 100 μM GEA, However there was a concentration-dependent reduction in fluorescence on addition of SB203580, with 50 μM producing a statistically significant effect, bringing caspase activity down to a level that is significantly different to 100 μM GEA 3162-treated cells in the absence of inhibitor, and not significantly different from untreated cells.

In contrast, inhibition of p38 enhanced STSP-induced apoptosis in Jaw II-GFP cells (Fig. 8A), with specific inhibition of p38 MAP kinase providing an additive apoptotic effect to the general kinase inhibition provided by STSP. Fluorescence levels were elevated in response to 200 nM STSP. Addition of 10 μM SB203580 in the presence of STSP increased fluorescence in a concentration-dependent manner, with 50 μM having a significant effect (*p* < 0.05 compared to STSP alone).

## 4. Discussion

Apoptotic cell death is regulated by NO and related species, such as ONOO^−^, in several cell types including myeloid-derived leukocytes such as neutrophils, eosinophils and monocytes/macrophages [10]. The biological effects of NO and ONOO−, including their effects on apoptosis, have recently been comprehensively reviewed [19]. Some studies have reported that p53 is required for apoptosis induced by NO and/or ONOO^−^ in some cell types [20-22]. Other studies suggest that p53 may be inactivated by ONOO^−^ [23] or that apoptosis may proceed despite the absence of p53 [24]. Alternatively, lack of p53 may confer a degree of resistance to NO/ONOO−-stimulated apoptosis but other pathways may contribute to cell death or compensate when p53 is lacking [25-28]. In order to elucidate the dependence or otherwise of apoptosis on p53, we investigated the effects of GEA 3162, a ONOO^−^ donor similar to SIN-1 [14], on apoptosis in a murine bone marrow cell line, Jaws II. This cell line is devoid of p53, and was studied containing either a vector encoding GFP alone (control) or a vector coding for both GFP and the anti-apoptotic protein, Bcl-2.

Although we have previously demonstrated that GEA 3162 simultaneously releases NO and O_2_^−^ in our system, and produces the same profile of apoptotic events as SIN-1 [14], other studies have found GEA 3162 to be a pure NO donor [29]. Furthermore, in culture conditions, factors such as CO_2_ may cause significant decay of ONOO^−^ formed [30]. Thus, although in this study we refer to GEA 3162 as a ONOO^−^ donor, the exact nature of the apoptogenic species is not known, and further studies (e.g. using SOD or NO scavengers) would have to be carried out to determine which species is responsible for these effects.

Apoptosis is a process that is dependent on the action of caspase proteases [1]. Therefore, total caspase activity was measured in Jaws II cells to determine whether these proteases are activated on exposure to GEA 3162, or STSP as a control apoptosis-initiating stimulus. STSP increased caspase activation in Jaws II-GFP cells, although this failed to reach statistical significance. GEA 3162 also increased caspase activity; the effect was clearly concentration-dependent and there was a significant effect with the higher concentration. When the pan-caspase inhibitor, zVAD, was added as an internal control for the assay, fluorescence was reduced in all conditions, demonstrating that fluorescence measured was due to caspase activation. A significant difference was seen between control cells exposed to 100 μM GEA 3162 in the absence and presence of zVAD, therefore GEA 3162 specifically enhances caspase activation. Thus, apoptosis proceeded despite the absence of p53 in these cells, and correlates with existing data that suggests that p53 is not essential for apoptosis, but alternative and/or compensatory pathways are involved.

Overexpression of Bcl-2 was protective against GEA 3162-induced caspase activation, as no significant increase in caspase activity was seen in Jaws II-Bcl-2 cells compared to untreated cells of the same kind. However, a significant difference was observed between treatment with 100 μM GEA 3162 in Jaws II-GFP compared with Jaws II-Bcl-2. Thus, it is the overexpressed Bcl-2 that abrogates caspase activity. Similar findings have previously been reported in macrophage cell lines, which were protected from NO-induced apoptosis by overexpression of Bcl-2 [31]. In that study, Bcl-2 was proposed to neutralise the p53-dependent increase in Bax expression. The absence of p53 in Jaws II cells implies that an alternative mechanism of protection is also involved. One possibility is the antioxidant activity of Bcl-2, which has been previously described [32]. Alternatively, Bcl-2 may have a protective role similar to that observed in *C. elegans* by its ortholog, CED-9. CED-9 acts to sequester CED-4 (the worm equivalent of Apaf-1), and prevent activation of CED-3 (caspase 3 in humans). Therefore, it is possible that human Bcl-2 may have a similar, more direct, role in caspase regulation, and there is some data to support this proposal, as discussed by Cory and Adams [4].

Mitochondrial permeability was enhanced in Jaws II-GFP cells on exposure to GEA 3162, suggesting that the mitochondrial pathway is involved in ONOO^−^ induced apoptosis in these cells. Bcl-2 overexpression reduced the change in *ψ*_M_ so that it was no longer significantly different from control, but this protection was incomplete. This is in agreement with previous studies in ONOO^−^ treated thymocytes, in which Bcl-2 offered complete protection against caspase activation and DNA fragmentation, but only partial inhibition of loss of *ψ*_M_ [33].

Measurement of specific caspases showed that GEA 3162 strongly activated caspases 2 and 3 in Jaws II-GFP cells, while a lesser activity of caspases 8 and 9 was detectable. Such data shows remarkable similarity to that obtained in HL-60 cells on exposure to ONOO^−^, in which activity of caspases 2 and 3 was approximately 500% of control (untreated) levels, whereas caspases 8 and 9 had a maximal activation of ∼200–210% of control [34]. Interestingly, HL60 cells are also p53 null, suggesting that this profile of caspase activity may occur in response to ONOO^−^ in cells lacking this gene. The lack of discernible phenotype in the caspase 2−/− mouse points towards a potential role for caspase 2 as a compensatory molecule activated in the absence of other pro-apoptotic proteins, such as p53. However, although it is possible that this is a cell-specific apoptotic pathway seen in cells lacking functional p53, a stimulus-specific effect in p53-replete cells in response to ONOO^−^ cannot be ruled out. Further experiments in other p53−/− and p53+/+ cells, plus siRNA and repletion studies would need to be carried out to distinguish cell-specific and stimulus-specific effects of ONOO^−^.

Caspase 3 is the principal effector caspase involved in the apoptotic cascade, and cleaves specific target proteins within the cell, such as PARP, PKC-δ, protein kinases and structural proteins [35]. However, little is known about the role of caspase 2 in apoptosis, as it has largely been ignored in investigations of the apoptotic process, due to the lack of phenotype in the knockout mouse. However, there have been several suggestions regarding its function (particularly that it acts just upstream of mitochondrial permeabilisation) and these proposed functions are reviewed by Troy and Shelanski [18]. A recent study also attributed caspase 2 function to an ER stress pathway of apoptosis, functioning as both an initiator and effector caspase [36]. As ER stress has been implicated in the apoptotic response to NO/ONOO^−^ in p53-deficient murine microglial cells [24], this is a feasible suggestion for the mechanism by which ONOO^−^ induces apoptosis in Jaws II cells.

Caspase 9 activation measured by the profiling assay confirmed the involvement of mitochondria in apoptosis elicited by GEA 3162 in Jaws II-GFP cells. Cleavage of the caspase 8 substrate at low levels suggests either non-specific cleavage by high levels of caspases 2 and 3, or a potential role for death receptor clustering that is amplified via the caspase cascade. It has been proposed that reactive oxygen species may induce ligand-independent death receptor clustering in lipid rafts and subsequent caspase activation [37]. It remains to be seen whether this mechanism has a role to play in GEA 3162-provoked apoptosis.

It is interesting to note that exogenous expression of human Bcl-2 does not simply reduce caspase activity, but abolishes it in both untreated and GEA 3162-exposed cells. This is similar to the ‘complete’ inhibition of apoptosis previously reported in various cell types on overexpression of Bcl-2 [33,38], and suggests that Bcl-2 may have a tighter regulatory role on NO/ONOO^−^-induced caspase activation than mere mitochondrial stability. Additionally, all caspases are equally affected by Bcl-2, rather than just those downstream of mitochondria (caspases 9 and 3) pointing to a more general inhibitory effect than insertion of Bcl-2 into mitochondrial membranes. If only mitochondrial stability was altered, it would be expected that caspases 2 and 8 would still be elevated in the Jaws II-Bcl-2 cells. These data therefore support the hypothesis that cytoprotection afforded by Bcl-2 occurs via a similar pathway to that observed in *C. elegans* by CED-9.

The role of MAP kinases in cells is complex and somewhat variable, with both pro- and anti-apoptotic effects being reported, and is seemingly dependent upon the cell type being investigated [7,8]. However, there is evidence to suggest that both p38 and JNK MAP kinases may play a role in NO or ONOO^−^ mediated cell death [39,40] as well as other apoptotic stimuli [41] and Bcl-2 has been shown to suppress p38 activation and subsequent NO-induced apoptosis [42], therefore it was hypothesised that these kinases may be involved in GEA 3162-induced apoptosis of Jaws II cells. Studies using pharmacological inhibitors of p38 and JNK demonstrated a pro-apoptotic role for p38 when Jaws II-GFP cells were exposed to ONOO^−^ from GEA 3162, but little or no role for JNK. However the role of p38 is stimulus-specific, as this kinase has an anti-apoptotic role when cells are exposed to STSP. Additional studies examining the expression and phosphorylation status of these MAP kinases would be useful in order to confirm and further investigate the involvement of these proteins and their role in ONOO^−^-induced apoptosis.

Protein modification may occur in response to reactive oxygen and nitrogen species, which may subsequently alter protein function and cause cellular effects including apoptosis [43]. One such modification, which may occur on exposure to ONOO^−^ is formation of 3-nitrotyrosine (3-NT), in which susceptible protein tyrosine residues may be nitrated [44]. Additional studies are required to assess formation of 3-NT, and identification of target proteins, in Jaws II cells exposed to GEA 3162, to determine whether this mechanism may have a role in GEA 3162-induced apoptosis in these cells.

We have demonstrated an apoptotic response to ONOO^−^ in a murine bone marrow cell line. To the best of our knowledge, this is the first time that apoptosis has been reported using this cell line, and demonstrates that functional p53 is not a requirement for ONOO^−^ mediated cell death in this cell type. Furthermore, we have shown that caspases 2 and 3 are important for apoptosis to proceed, with roles for caspases 8 and 9, and p38 MAP kinase. The anti-apoptotic protein, Bcl-2, abolishes caspase activation involved in both intrinsic and extrinsic pathways, suggesting a survival mechanism that is not restricted solely to the mitochondria, and may represent a novel or previously only speculative mechanism by which Bcl-2 protects against apoptosis.

## Abbreviations

GEA 3162: 1,2,3,4-oxatriazolium,5-amino-3-(3,4-dichlorophenyl)-chloride
STSP: staurosporine
GFP: green fluorescent protein
Jaws II-GFP: p53-deficient murine bone marrow cells stably transfected with a vector encoding GFP
Jaws II-Bcl-2: p53-deficient murine bone marrow cells stably transfected with a vector encoding GFP and the survival protein Bcl-2
JNK: c-Jun N-terminal kinase
MAP kinase: mitogen activated protein kinase
NO: nitric oxide
ONOO^−^: peroxynitrite
